# Scabies Infestation and Risk of Acute Myocardial Infarction: A Population-Based Cohort Study

**DOI:** 10.3390/jpm12020229

**Published:** 2022-02-07

**Authors:** Yao-Ping Ko, Pei-Yun Chen, Chung Y. Hsu, Renin Chang, Kai-Chieh Hu, Lu-Ting Chiu, Yao-Min Hung, Guang-Yuan Mar

**Affiliations:** 1 Department of Emergency Medicine, Kaohsiung Veterans General Hospital, Kaohsiung 813414, Taiwan; metalman3747@gmail.com (Y.-P.K.); rhapsody1881@gmail.com (R.C.); 2 Department of Medical Education and Research, Kaohsiung Veterans General Hospital, Kaohsiung 81362, Taiwan; smp960133@gmail.com; 3 Department of Physical Medicine and Rehabilitation, Kaohsiung Armed Forces General Hospital, Kaohsiung 80284, Taiwan; 4 Graduate Institute of Biomedical Sciences, China Medical University, Taichung 40402, Taiwan; hsucy63141@gmail.com; 5 Management Office for Health Data, China Medical University Hospital, Taichung 40402, Taiwan; billyhu.cmuh@gmail.com (K.-C.H.); luting.cmuh@gmail.com (L.-T.C.); 6 College of Medicine, China Medical University, Taichung 40402, Taiwan; 7 Department of Internal Medicine, Kaohsiung Municipal United Hospital, Kaohsiung 80457, Taiwan; ymhung1@gmail.com; 8 College of Health and Nursing, Meiho University, Pingtung 91202, Taiwan; 9 Institute of Medicine, Chung Shan Medical University, Taichung 40201, Taiwan

**Keywords:** scabies, acute myocardial infarction, cohort study, NHIRD

## Abstract

Background: Scabies is an infectious inflammatory skin disease. Cytokine-mediated inflammatory responses may be one of the pathological mechanisms underlying myocardial infarction. Objective: We explore the association between scabies and subsequent acute myocardial infarction (AMI) and all-cause mortality; Methods: We conducted a nationwide population-based study using data from the National Health Insurance Research Database (NHIRD) in Taiwan. Patients with scabies (*n* = 30,184) and 120,739 controls without scabies were included. The primary outcomes were incidental AMI and all-cause mortality. Using Cox proportional-hazards regression analysis, we estimated the risk of acute myocardial infarction for the study cohort; Results: The mean age of the study cohort was 51.81 ± 19.89 years. The adjusted sub-distribution hazard ratios (aSHRs) of AMI were 1.214 (95% CI, 1.068–1.381) after adjusting for demographic characteristics, income, OPD utility frequency, days in hospital, co-morbidities, and medication. The adjusted hazard ratio (aHR) of all-cause mortality after adjusting for age, gender, income, OPD utility frequency, days in hospital, co-morbidities, co-medication, and urbanization was 1.612 (95% CI, 1.557–1.669). Conclusions: Our study showed that patients with scabies infestations were at higher risk for subsequent AMI and all-cause mortality.

## 1. Introduction

Scabies is an itchy, highly contagious parasitic disease of the skin caused by *Sarcoptes scabiei*. The global prevalence of scabies is about 300 million cases per year, placing a heavy burden on infected individuals and health care systems, regardless of the socioeconomic level of the country where scabies cases are found [1,2]. Most patients with scabies, i.e., 90–99%, present with pruritis [3]. It decreases the quality of life of infected patients and may lead to secondary infections. Previous studies have identified scabies-related complications, including chronic kidney disease (CKD) and chronic obstructive pulmonary disease (COPD) [2,4,5]. A nationwide study reported an increased risk of stroke in patients with scabies and suggested that this association may be due to immunopathological factors [6]. Immune and inflammatory responses are the basic pathophysiological mechanisms underlying scabies infections [7]. According to previous studies, pro-inflammatory cytokines, including interleukin (IL)-1, IL-6, IL-8, and TNF-α, as well as immunomodulatory cytokines IL-10 and IL-12, are responsible for the immune response to scabies infections [8]. A severe form of scabies infection in humans may be associated with an IgE-driven Th2 response [9]. Further evidence reveals a complex immune response to scabies infestation. A porcine model of scabies infection suggests that IL-17-related pathways may contribute to the immunopathology of crusted scabies. The inflammatory response and the production of cytokines, such as IL-17, are important factors in many inflammatory diseases [10,11]. Atherosclerosis develops as a result of chronic inflammation and triggers ischemic events that cause a strong acute inflammatory response [12]. Acute myocardial infarction (AMI) is caused by the sudden necrosis of many cardiomyocytes, resulting in the release of their intracellular contents. AMI is defined as a clinical or pathological event in the presence of myocardial ischemia with signs of myocardial injury [13]. In addition, AMI is recognized as the leading cause of cardiovascular disease (CVD) morbidity and mortality worldwide [14]. Inflammation is widely believed to play a central role in the pathogenesis of atherosclerosis and acute coronary events [15,16]. 

In this study, we hypothesized that scabies infestation provides better conditions for the release of IL-17 or other cytokines and induces the immunopathological response of AMI. However, no studies investigating the epidemiological relationship between scabies infestation and the subsequent occurrence of AMI and all-cause mortality have been reported. Therefore, we conducted this retrospective nationwide cohort study to explore this issue.

## 2. Materials and Methods

### 2.1. Data Source

The Taiwan National Health Insurance is a universal health insurance system covering >99% of the 23 million Taiwan residents. We conducted a nationwide cohort study by mining the National Health Insurance Research Database (NHIRD), which collects beneficiaries’ registration files regarding demographics, all types of medical visits, laboratory tests codes, procedure codes, prescription codes, and diagnostic codes based on the International Classification of Diseases, Ninth Revision, Clinical Modification (ICD-9-CM) and the International Classification of Diseases, Tenth Revision, Clinical Modification (ICD-10-CM). Prior to publishing the database for the study, the original ID numbers were anonymized to protect the privacy of the patients. The study was approved by the Institutional Review Board of the China Medical University Hospital Research Ethics Committee (CMUH109-REC2-031), which waived the requirement for informed consent because data in the NHIRD are anonymized. 

### 2.2. Study Subjects

The study subjects were selected from the NHIRD dataset. We selected patients who were newly diagnosed with scabies (ICD-9-CM code: 133.0) from January 2000 to December 2016. The diagnosis was made by a practicing physician based on the patient’s medical history and physical examination findings. The index date was defined as the first date that scabies was diagnosed. Only patients with at least one inpatient admission or two outpatient visits with a scabies diagnosis were selected. Exclusion criteria for the study cohort were as follows: (1) patients diagnosed before 1 January 2000; (2) patients younger than 20 years of age; (3) patients with a previous history of scabies or AMI; and (4) patients with a concurrent diagnosis of scabies, AMI, and death. A total of 30,184 subjects with newly diagnosed scabies infestations were identified from the NHIRD for the study based on the above criteria. The control group was selected from the NHIRD, randomized, and matched at a ratio of 1:4 by age, gender, index year, low income status, hospital days, outpatient department (OPD) utility frequency, urbanization, co-morbidities, and co-medications list in Table 1. Finally, 120,739 subjects were enrolled as a comparison cohort (non-scabies group). Individuals in both the study group and the control group were followed up until there was an AMI event, until December 2017, or until death. The death of an individual was defined by a record in the death certificate database of Taiwan.

### 2.3. Outcome and Covariates

All ambulatory medical care and inpatient medical records for subjects in both groups were tracked from the index date through the end of 2017. The main outcomes of the study were AMI (ICD-9-CM code: 410 and ICD-10-CM code: I21) and all-cause mortality. Both cohorts were followed up until AMI, all-cause mortality, patient withdrawal from the NHI program, or the end of 2017, whichever occurred first. Validation of ICD codes for the diagnosis of AMI have been done in previous studies [17]. For further refinement, only patients with at least one inpatient admission or more than two ambulatory visits with the diagnosis of scabies were eligible. We classified age into four groups: <50, 50–64, 65–74, and >75 years. The co-morbidities analyzed in this study were diabetes (ICD-9-CM code: 250 and ICD-10-CM codes: E10–14), hypertension (ICD-9-CM codes: 401–405 and ICD-10-CM codes: I10, I15), hyperlipidemia (ICD-9-CM code: 272 and ICD-10-CM code: E78), coronary artery disease (CAD) (ICD-9-CM codes: 410–414 and ICD-10-CM codes: I20–25), cancer (ICD-9-CM codes: 140–208 and ICD-10-CM codes: C00–C96), chronic kidney disease (CKD) (ICD-9-CM codes: 584–586 and ICD-10-CM code: N18), chronic obstructive pulmonary diseases (COPD) (ICD-9-CM codes: 491, 492, 496, and ICD-10-CM code: J44), sleep apnea (ICD-9-CM codes: 327.23, 780.51, 780.53, 780.57, and ICD-10-CM codes: G47.30, G47.33, G47.39), atrial fibrillation (ICD-9-CM code: 427.3 and ICD-10-CM code: I48), heart failure (ICD-9-CM code: 428 and ICD-10-CM code: I50), smoking, peripheral arterial obstructive disease (PAOD) (ICD-9-CM code: 443 and ICD-10-CM code: I70.2), Parkinson’s disease (ICD-9-CM code: 332.0 and ICD-10-CM code: G20), ischemic stroke (ICD-9-CM codes: 433, 434, 436, 437 and ICD-10-CM code: I63), human immunodeficiency virus (HIV), liver cirrhosis (ICD-9-CM code: 571 and ICD-10-CM code: K70, K72, K73, K74), inflammatory bowel disease (IBD) (ICD-9-CM code: 555, 556 and ICD-10-CM code: K50, K51), systemic lupus erythematosus (SLE) (ICD-9-CM code: 710.0 and ICD-10-CM code: M32), and rheumatoid arthritis (RA) (ICD-9-CM code: 714.0, 714.3 and ICD-10-CM code: M05.7–M05.9, M06.0, M06.2–M06.3, M06.8–M06.9, M08). Information on co-morbidities was obtained by tracking all ambulatory medical care and inpatient records in the National Health Insurance database from 1996 to 2000. Since an actively health-care-seeking individual will probably have a higher chance of being found positive for scabies, we also adjusted the frequency of outpatient visits identified within 2 years before the index date to control for potential selection bias. In addition, potential medication confounders in this study were aspirin, clopidogrel, ticlopidine, warfarin, metformin, statin, steroid, and colchicine.

### 2.4. Statistical Analysis

First, the distributions of age, gender, low income status, OPD utility frequency, days in hospital, co-morbidities, co-medication use, and urbanization level were compared between the scabies and the non-scabies groups using a 2-sample *t* test for continuous variables and a Chi square (χ^2^) test for categorical variables. A standardized mean difference (SMD) of 0.1 or less indicates a negligible difference [18]. Second, in both groups, the incidence of AMI and all-cause mortality was calculated as the number of patients with events detected during follow-up divided by the total follow-up time of 1000 person-years. We used multivariable Cox proportional-hazards regression models to estimate the association of scabies on hazard ratios (HRs) accompanied by 95% confidence intervals (CIs). The covariates used in the multivariable models included gender, age, low income, hospital days, OPD utility frequency, co-morbidity, co-medication, and urbanization listed in Table 1. Since death is a potential confounder that may bias the estimated risk of AMI, we analyzed a competing risk of death model to estimate the sub-hazard ratio and 95% CI for the incidence of AMI in both groups. Third, the Kaplan–Meier method was adopted to describe the cumulative incidence of AMI and all-cause mortality in the two groups by the log-rank test. All statistical analyses were carried out with SAS (Version 9.4; SAS Institute Inc., Cary, NC, USA). The level of statistical significance was set at *p* < 0.05 for a two-sided test. 

### 2.5. Sensitivity Analysis

To validate the robustness of the study findings, we conducted sensitivity analyses for both results, including AMI and all-cause mortality. Scabies diagnoses were limited to those made by board-certified dermatologists.

## 3. Results

After age-and sex-matching in a 1:4 ratio (one study patient to four control patients), a total of 150,923 patients were enrolled in the study, including 30,184 scabies patients and 120,739 control patients. The mean age of the scabies patients was 51.81 ± 19.89 years. The demographic characteristics of the two groups are listed in Table 1. There was a higher proportion of patients with longer hospital days in the scabies cohort. Other characteristics such as age, gender, low income, OPD utility frequency, co-morbidities, co-medication, and urbanization all show non-statistical difference in both groups.

The association between scabies and AMI is shown in Table 2. After adjusting for all covariates listed in Table 1 and considering death as a competing event, patients with scabies had a higher risk of AMI than controls with an adjusted sub-hazard ratio (aSHR) of 1.214 (95% confidence interval (CI), 1.068–1.381). Among the covariates, the aSHR was higher in older male subjects with co-morbidities and patients using medication. 

Table 3 shows the association between scabies and all-cause mortality. After adjusting for age, sex, low income, OPD utility frequency, days in the hospital, co-morbidity, co-medication, and urbanization, the adjusted HR (aHR) of scabies patients relative to the non-scabies group was 1.612 (95% CI, 1.557–1.669; *p* < 0.001). 

Table 4 demonstrates the results of stratification analysis. After adjusting for age, sex, low income, OPD utility frequency, days in the hospital, co-morbidity, co-medication, and urbanization and using the competing risk model of death, the scabies group had a higher risk of AMI in patients aged 65–74 (aSHR, 1.393; 95% CI, 1.093–1.774; *p* < 0.01) compared with the age-matched group controls. However, the interaction by age was not significant (*p* = 0.1512). In the analysis of subgroup by gender, female scabies patients had a higher risk of AMI (aSHR, 1.376; 95% CI, 1.140–1.659) than male scabies patients (aSHR, 1.077; 95% CI, 0.902–1.286). The interaction by gender was significant (*p* = 0.0007).

Table 4 also displays the association between scabies and all-cause mortality in different stratifications. Age, gender, and co-morbidity acted as effect modifiers. In the age subgroup analysis, compared with the non-scabies matched group, the all-age group had a higher risk of all-cause mortality (aHR 1.402 in aged <50; aHR 1.692 in aged 50–64; aHR 1.711 in aged 65–74; aHR 1.543 in aged >75; all *p* < 0.001). In the income subgroup analysis, the non-low-income group with scabies showed risk of all-cause mortality (aHR, 1.619; 95% CI, 1.563–1.677), and the low-income group with scabies also did. (aHR, 1.265; 95% CI, 1.033–1.549). 

Table 5 and Table 6 show the results of sensitivity analyses. The diagnosis of scabies was limited to those made by board-certified dermatologists. Even under stricter definitions of scabies by licensed dermatologists, we still found a higher risk of AMI in the scabies group compared with the non-scabies group (aHR, 1.200; 95% CI, 1.063–1.356) and in all-cause mortality (aHR, 1.362; 95% CI, 1.320–1.406). 

**Figure 1:**
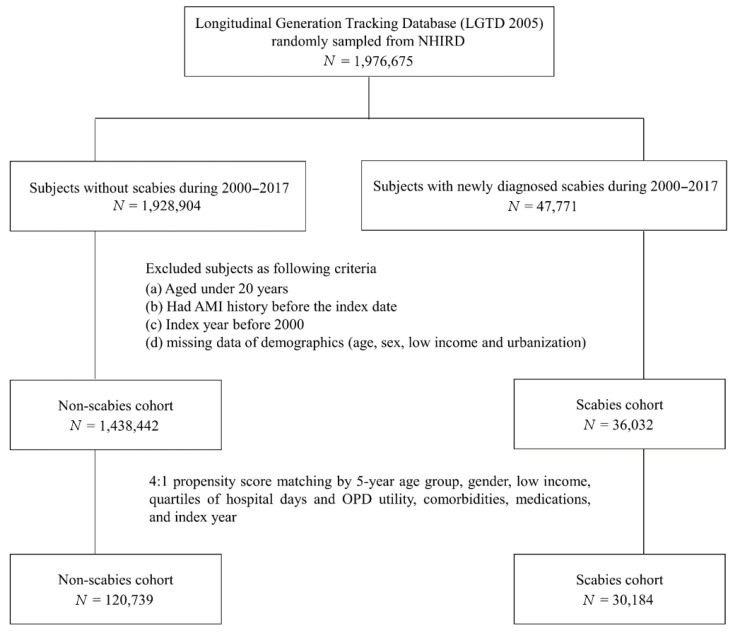
Flow chart of the study population selection.

**Figure 2:**
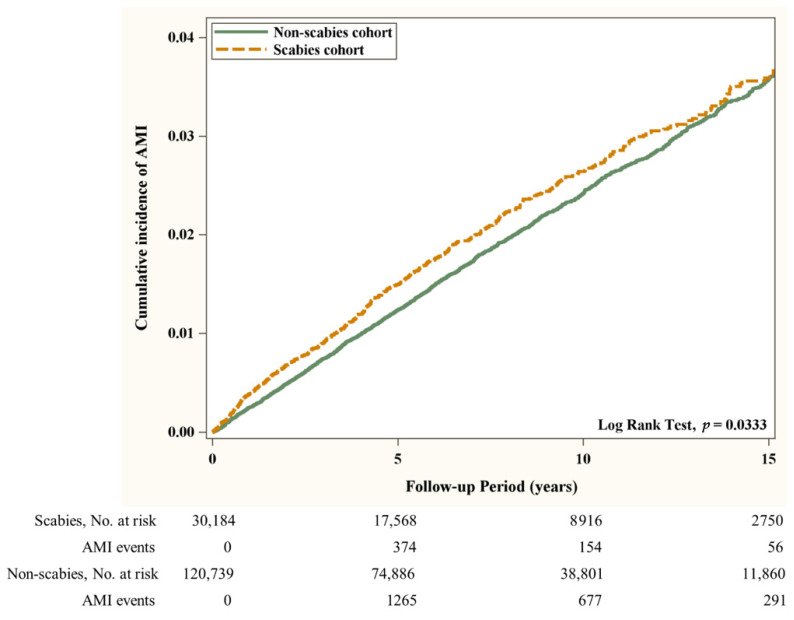
Cumulative incidence of AMI in patients with and without scabies. Abbreviation: (AMI), Acute Myocardial Infarction.

**Figure 3:**
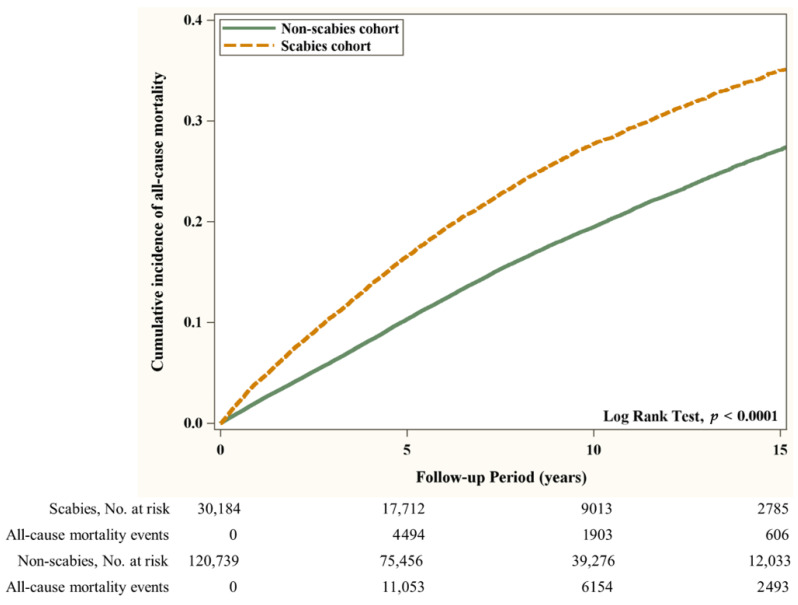
Cumulative risk of all-cause mortality in patients with and without scabies.

Flow chart of the study population selection is presented in Figure 1. In Figure 2, the Kaplan–Meier curve demonstrates the cumulative incidence of AMI tested by the log-rank test. An increased risk of AMI was observed in the scabies group compared with the non-scabies group (*p* = 0.0333). Figure 3 displays the increased cumulative risk of all-cause mortality in the scabies group compared with the controls (*p* < 0.001).

## 4. Discussion

To our knowledge, this is the first nationwide population-based cohort study to investigate the risk between scabies and subsequent AMI. Compared with patients without scabies, patients with scabies demonstrate a higher incidence rate of subsequent AMI at 2.70 per 1000 person-years (aSHR, 1.214; 95% CI, 1.068–1.381). Meanwhile, the incidence rate of all-cause mortality is also higher in patients with scabies compared with patients without scabies (aHR, 1.612; 95% CI, 1.557–1.669). This finding suggests that scabies may be an independent risk factor for AMI.

The subjects enrolled in our study were collected from the NHIRD dataset. As shown in Table 1, the age distribution of the scabies group was mostly above 50 years. The above distribution is similar to those of previous scabies studies using the NHIRD dataset [19]. Co-morbidities, including hypertension, diabetes, CKD, COPD, heart failure, Parkinson’s diseases, ischemic stroke, HIV, liver cirrhosis, IBD, SLE, and RA were near the same prevalence in the scabies group and in the non-scabies group. These finding suggest that the co-morbidities were well balanced in both groups in our study. In addition, co-medications were also well matched in both groups. 

Inflammation plays a key role in coronary artery disease because it can cause atherosclerotic lesions in human arteries [20]. Chemokines and the activation of monocytes and T cells, which enhance the localized inflammatory response, lead to chemotaxis and the accumulation of macrophages in fatty streaks in atherosclerotic plaques, resulting in the progression of atherosclerosis [15]. Activated macrophages, T cells, and mast cells at the sites of atherosclerotic plaque rupture produce several types of molecules, such as inflammatory cytokines, which can destabilize lesions [20]. Finally, the ruptured plaque may lead to acute thrombosis and ischemia, which is the pathogenic mechanism of AMI. According to previous studies, infection in the human body generates circulating inflammatory cytokines, such as IL-1, -6, and -8 and TNF-α, which can activate inflammatory cells in atherosclerotic plaques to destabilize them [21]. This mechanism has been proven in that an increased risk of myocardial infection exists beyond the short-term post-infection period of infections, such as mild respiratory infections, urinary tract infections, pneumonia, and pneumonia complicated by sepsis [21]. As we know, infection and inflammation influence the risk of myocardial infarction. 

Our results supported our hypothesis that scabies infections may influence the progression of atherosclerosis and lead to subsequent AMI. Previous studies have shown that patients with scabies have an elevated risk of stroke, which is also a result of atherosclerosis [6]. Scabies is a skin infestation caused by the mite *Sarcoptes scabiei*; it is not only a simple, itchy skin disease but can activate many serious downstream systemic and life-threatening diseases [22]. In previous studies, scabies has been linked to secondary complications, such as rheumatic heart disease (RHD) and acute post-streptococcal glomerulonephritis (APSGN), via the immune response caused by the disease itself and other associated bacterial infections [22,23]. Scabies can be divided into two forms, common scabies and severe crusted scabies, each associated with different protective immune responses of the host [24,25]. Current data suggest that the immune response to common scabies is dominated by Th1 cells and their cytokine profile, while the response to crusted scabies is dominated by Th2 cells [24,25]. Otherwise, keratinocytes, Langerhans cells, and macrophages in the skin all respond to the mite antigens, secreting pro-inflammatory cytokines such as TNF-α, IFN-γ, TGF-β, and IL-23. This leads to the differentiation of CD8 + T and CD4 + Th1 and Th2 cells and their recruitment to the skin. Cytokines secreted in these mechanisms include IL-1, IL-4, IL-5, IL-6, IL-8, TNF-α, IL-10, IL-13, and IFN-γ [26,27,28,29]. A porcine model of scabies infection suggests that IL-17-related pathways may contribute to the immunopathology of crusted scabies [30]. IL-1, IL-6, TNF-α, and IL-17 can activate the inflammatory response in atherosclerotic plaques and destabilize the lesions, which increases the possibility of subsequent AMI [26,31]. However, all the above mechanisms associated with scabies and AMI are potential hypotheses that need further basic and large-scale clinical studies to confirm. 

In our study, the incidence rate of AMI increased with age in both the non-scabies and scabies groups. This finding is consistent with the results of previous studies, which also used the NHIRD in Taiwan as the data source [32,33]. Our findings suggest that the risk of AMI was higher in the scabies group than in the non-scabies group, especially among the elderly (age groups 65–74 years and >75 years). This finding may encourage clinicians to consider the prevention of subsequent AMI in older patients with a history of scabies. The most important in our study is that the scabies group had a higher risk of AMI than in the non-scabies group, independent of age, gender, income, co-morbidities, and co-medication. In addition, our study showed that the scabies group had a higher risk of all-cause mortality than the non-scabies group, independent of age, gender, income, co-morbidities, and co-medication.

Our study design used ICD-9-CM and ICD-10-CM codes to define the primary outcome of AMI. This design appears to be effective, as a previous study has shown positive predictive values (PPVs) from 0.88 to 0.90 using ICD codes as diagnoses of AMI [17]. The strength of our study is that it is a novel retrospective cohort study using large amounts of nationwide data to assess the risk of AMI in patients with scabies. 

There are several limitations to our study. First, we did not have information on body mass index, lifestyle, and alcohol consumption, which may have influenced the incidence of AMI. Second, misclassification may have occurred because some patients with scabies have mild symptoms and do not seek medical assistance.

## 5. Conclusions

Patients with scabies infestations have an increased risk of subsequent AMI and all-cause mortality. Our findings may alert clinicians to this possibility in patients with scabies and give more attention to the signs or symptoms associated with AMI in this population.

## Figures and Tables

**Table 1:** Baseline demographic factors, co-morbidities, and medications between scabies and non-scabies cohorts.

	Non-Scabies Cohort ^#^		Scabies Cohort		
Characteristics ^	(*n* = 120,739)		(*n =* 30,184)		
	*n*	%	*n*	%	SMD ^&^
Age, years					
<50	58,257	48.25	14,418	47.77	0.0097
50–64	27,625	22.88	6892	22.83	0.0011
65–74	14,789	12.25	3728	12.35	0.0031
75+	20,068	16.62	5146	17.05	0.0114
Mean ± SD	51.48 ± 19.77		51.81 ± 19.89		0.0166
Gender					
Female	52,777	43.71	13,592	45.03	0.0265
Male	67,962	56.29	16,592	54.97	0.0265
Low income	3711	3.07	981	3.25	0.0101
Hospital days	21.45 ± 43.77		29.38 ± 53.65		0.1621
OPD utility frequency	45.87 ± 40.37		48.81 ± 44.47		0.0694
Comorbidity					
Diabetes	25,196	20.87	6485	21.48	0.0151
Hypertension	46,828	38.78	11939	39.55	0.0158
Hyperlipidemia	31,258	25.89	7858	26.03	0.0033
Coronary artery disease	25,070	20.76	6426	21.29	0.0129
Cancer	4392	3.64	1128	3.74	0.0053
Chronic kidney disease	8405	6.96	2223	7.36	0.0156
COPD	32,192	26.66	8150	27.00	0.0076
Sleep apnea	857	0.71	229	0.76	0.0057
Atrial fibrillation	2941	2.44	743	2.46	0.0017
Heart failure	7888	6.53	2072	6.86	0.0133
Smoking	5484	4.54	1315	4.36	0.0090
PAOD	3639	3.01	960	3.18	0.0096
Parkinson	3282	2.72	948	3.14	0.0251
Ischemic stroke	18,929	15.68	4834	16.02	0.0092
HIV	251	0.21	72	0.24	0.0065
Liver cirrhosis	2953	2.45	793	2.63	0.0115
IBD	9	0.01	3	0.01	0.0027
SLE	130	0.11	29	0.10	0.0036
RA	293	0.24	80	0.27	0.0044
Co-medication use > 90 days before index date					
Aspirin	18,710	15.50	4809	15.93	0.0120
Clopidogrel	2299	1.90	634	2.10	0.0140
Ticlopidine	255	0.21	83	0.27	0.0130
Warfarin	1274	1.06	323	1.07	0.0015
Metformin	13,075	10.83	3375	11.18	0.0113
Statin	12,251	10.15	3156	10.46	0.0102
Steroid	11,903	9.86	3056	10.12	0.0089
Co-medication use > 30 days before index date					
Colchicine	6991	5.79	1836	6.08	0.0124
Urbanization ^$^					
1 (high)	64,110	53.10	15649	51.85	0.0251
2	45,504	37.69	11512	38.14	0.0093
3	9310	7.71	2569	8.51	0.0293
4 (low)	1815	1.50	454	1.50	0.0001

^: Data shown as *n* (%) or mean ± SD. ^$^: The urbanization level was categorized by the population density of the residential area into four levels, with level 1 as the most urbanized and level 4 as the least urbanized. ^#^: Using 4:1 propensity score matching. ^&^: SMD, standardized mean difference. A standardized mean difference of 0.1 or less indicates a negligible difference. Abbreviation: (COPD), Chronic Obstructive Pulmonary Disease; (HIV), Human Immunodeficiency Virus; (IBD), Inflammatory Bowel Disease; (OPD), Out Patient Department; (RA), Rheumatoid Arthritis; (PAOD), peripheral Arterial Occlusion Disease; (SLE), Systemic Lupus Erythematosus.

**Table 2:** HRs and 95% CIs of AMI associated with scabies and other covariates (competing risk model of death).

Variables	Obs.	AMI (*n* = 2859)			Crude HR (95% CI)	Adjusted ^$^ SHR (95% CI)
		Event	PY	IR ^		
Scabies						
No	120,739	2267	926,228.75	2.45	ref.	ref.
Yes	30,184	592	219,000.65	2.70	1.105 (1.009–1.210) *	1.214 (1.068–1.381) **
Age, years						
<50	72,675	379	645,195.37	0.59	ref.	ref.
50–64	34,517	739	252,355.04	2.93	5.081 (4.487–5.754) ***	2.549 (2.069–3.139) ***
65–74	18,517	695	124,866.86	5.57	9.678 (8.532–10.978) ***	3.309 (2.673–4.096) ***
75+	37,054	1046	122,812.14	8.52	15.186 (13.469–17.121) ***	4.465 (3.628–5.496) ***
Gender						
Female	66,369	999	522,991.57	1.91	ref.	ref.
Male	84,554	1860	622,237.84	2.99	1.555 (1.439–1.680) ***	1.312 (1.180–1.459) ***
Low income						
No	146,231	2751	1,109,055.55	2.48	ref.	ref.
Yes	4692	108	36,173.86	2.99	1.197 (0.985–1.453)	1.377 (1.016–1.868) *
Hospital days					1.000 (0.999–1.001)	1.000 (0.999–1.001)
OPD utility frequency					1.009 (1.009–1.010) ***	1.000 (0.999–1.001)
Comorbidity						
No	59,264	292	546,924.18	0.53	ref.	ref.
Yes	91,659	2567	598,305.22	4.29	8.159 (7.220–9.220) ***	2.814 (2.104–3.765) ***
Co-medication						
No	103,066	1063	880,094.78	1.21	ref.	ref.
Yes	47,857	1796	265,134.62	6.77	5.837 (5.397–6.311) ***	2.242 (1.969–2.552) ***
Urbanization						
1 (high)	79,759	1416	614,083.48	2.31	ref.	ref.
2	57,016	1088	426,997.32	2.55	1.110 (1.025–1.202) *	0.955 (0.855–1.066)
3	11,879	282	87,797.79	3.21	1.397 (1.229–1.589) ***	0.972 (0.807–1.170)
4 (low)	2269	73	16,350.82	4.46	1.934 (1.526–2.450) ***	1.089 (0.764–1.552)

*: *p* < 0.05, **: *p* < 0.01, ***: *p* < 0.001. ^: IR, Incidence Rate, per 1000 person-years. ^$^: Adjusted for age, gender, low income, OPD utility frequency, hospital days, co-morbidity, co-medication, and urbanization. Abbreviation: (AMI), Acute Myocardial Infarction; (CI), Confidence Interval; (SHR), Sub-Hazard Ratio; (Obs), Observed number of patients; (OPD), Out Patient Department; (PY), Person-Year.

**Table 3:** HRs and 95% CIs of all-cause mortality associated with scabies and other covariates.

Variables	Obs.	All-Cause Mortality (*n* = 27,105)			Crude HR (95% CI)	Adjusted ^$^ HR (95% CI)
		Event	PY	IR ^		
Scabies						
No	120,739	20,021	932,755.58	21.46	ref.	ref.
Yes	30,184	7084	220,519.65	32.12	1.496 (1.456–1.537) ***	1.612 (1.557–1.669) ***
Age, years						
<50	72,675	2513	646,787.53	3.89	ref.	ref.
50–64	34,517	4414	255,018.13	17.31	4.602 (4.382–4.833) ***	2.449 (2.289–2.621) ***
65–74	18,517	6222	126,677.97	49.12	13.212 (12.613–13.841) ***	4.552 (4.257–4.868) ***
75+	25,214	13,956	124,791.6	111.83	31.626 (30.292–33.019) ***	8.812 (8.267–9.393) ***
Gender						
Female	66,369	10,788	525,496.96	20.53	ref.	ref.
Male	84,554	16,317	627,778.27	25.99	1.262 (1.232–1.293) ***	1.296 (1.257–1.337) ***
Low income						
No	146,231	26,002	1,116,787.04	23.28	ref.	ref.
Yes	4692	1103	36,488.19	30.23	1.299 (1.223–1.379) ***	1.382 (1.262–1.513) ***
Hospital days					1.003 (1.003–1.003) ***	1.004 (1.004–1.004) ***
OPD utility frequency					1.009 (1.009–1.010) ***	1.000 (1.000–1.001)
Co-Morbidity						
No	59,264	2400	548,208.97	4.38	ref.	ref.
Yes	91,659	24,705	605,066.26	40.83	9.501 (9.109–9.910) ***	2.715 (2.497–2.953) ***
Co-Medication						
No	103,066	11,356	883,969.76	12.85	ref.	ref.
Yes	47,857	15,749	269,305.47	58.48	4.685 (4.571–4.803) ***	1.310 (1.265–1.357) ***
Urbanization						
1 (high)	79,759	12,661	618,189.34	20.48	ref.	ref.
2	57,016	11,198	429,958.82	26.04	1.270 (1.238–1.303) ***	1.036 (1.003–1.071) *
3	11,879	2702	88,565.16	30.51	1.487 (1.427–1.550) ***	1.035 (0.980–1.092)
4 (low)	2269	544	16,561.91	32.85	1.600 (1.469–1.744) ***	0.961 (0.860–1.074)

*: *p* < 0.05, ***: *p* <0.001. ^: IR, Incidence Rate, per 1000 person-years. ^$^: Adjusted for age, gender, low income, OPD utility frequency, hospital days, co-morbidity, co-medication, and urbanization. Abbreviation: (CI), Confidence Interval; (HR), Hazard Ratio; (Obs), Observed number of patients; (OPD), Out Patient Department; (PY), Person-Year.

**Table 4:** HRs and 95% CIs of AMI and all-cause mortality stratified by age, gender, low income, co-morbidity, co-medication, and urbanization.

	Obs.	Non-Scabies Cohort			Obs.	Scabies Cohort			Crude HR (95% CI)	Adjusted SHR ^$^ (95% CI)	*p* for Interaction
Variables		Event	PY	IR ^		Event	PY	IR ^			
AMI (competing risk model of death)											
Age group											0.1512
<50	58,257	297	518,198.26	0.57	14,418	82	126,997.11	0.65	1.112 (0.870–1.422)	1.307 (0.874–1.955)	
50–64	27,625	592	204,681.49	2.89	6892	147	47,673.54	3.08	1.074 (0.896–1.288)	1.006 (0.768–1.318)	
65–74	14,789	534	102,243.46	5.22	3728	161	22,623.40	7.12	1.409 (1.181–1.682) ***	1.393 (1.093–1.774) **	
75+	20,068	844	101,105.54	8.35	5146	202	21,706.60	9.31	1.111 (0.951–1.298)	1.182 (0.961–1.452)	
Gender											0.0007
Female	52,777	752	421,210.25	1.79	13,592	247	101,781.32	2.43	1.353 (1.171–1.563) ***	1.376 (1.140–1.659) ***	
Male	67,962	1515	505,018.51	3.00	16,592	345	117,219.33	2.94	0.984 (0.874–1.106)	1.077 (0.902–1.286)	
Low income											0.2305
No	117,028	2177	897,297.89	2.43	29,203	574	211,757.65	2.71	1.117 (1.018–1.225) *	1.211 (1.062–1.379) **	
Yes	3711	90	28,930.86	3.11	981	18	7243.00	2.49	0.818 (0.493–1.358)	1.242 (0.624–2.473)	
Comorbidity											0.6504
No	47,533	232	439,496.92	0.53	11,731	60	107,427.26	0.56	1.067 (0.803–1.418)	1.644 (0.896–3.016)	
Yes	73,206	2035	486,731.83	4.18	18,453	532	111,573.39	4.77	1.143 (1.038–1.258) **	1.193 (1.047–1.361) **	
Co-medication											0.5948
No	82,556	842	710,126.77	1.19	20,510	221	169,968.01	1.30	1.099 (0.947–1.276)	1.120 (0.868–1.444)	
Yes	38,183	1425	216,101.98	6.59	9674	371	49,032.64	7.57	1.155 (1.030–1.296) *	1.238 (1.067–1.436) **	
Urbanization											0.7785
1 (high)	64,110	1135	498,685.06	2.28	15,649	281	115,398.42	2.44	1.057 (0.926–1.206)	1.155 (0.956–1.397)	
2	45,504	855	344,151.52	2.48	11,512	233	82,845.80	2.81	1.146 (0.991–1.325)	1.293 (1.061–1.574) *	
3	9310	217	69,990.40	3.10	2569	65	17,807.39	3.65	1.188 (0.900–1.568)	1.215 (0.811–1.823)	
4 (low)	1815	60	13,401.77	4.48	454	13	2949.05	4.41	0.997 (0.546–1.817)	0.915 (0.344–2.432)	
All-Cause Mortality											
Age group											0.0418
<50	58,257	1855	519,481.71	3.57	14,418	658	127,305.83	5.17	1.448 (1.325–1.583) ***	1.402 (1.240–1.585) ***	
50–64	27,625	3203	206,868.68	15.48	6892	1211	48,149.46	25.15	1.632 (1.527–1.743) ***	1.692 (1.558–1.837) ***	
65–74	14,789	4546	103,671.59	43.85	3728	1676	23,006.38	72.85	1.695 (1.603–1.793) ***	1.711 (1.595–1.836) ***	
75+	20,068	10,417	102,733.62	101.40	5146	3539	22,057.98	160.44	1.613 (1.553–1.676) ***	1.543 (1.468–1.621) ***	
Gender											<0001
Female	52,777	7622	423,182.16	18.01	13,592	3166	102,314.80	30.94	1.713 (1.643–1.785) ***	1.612 (1.530–1.698) ***	
Male	67,962	12,399	509,573.43	24.33	16,592	3918	118,204.84	33.15	1.362 (1.314–1.412) ***	1.585 (1.512–1.660) ***	
Low income											0.0785
No	117,028	19,193	903,559.07	21.24	29,203	6809	213,227.98	31.93	1.502 (1.461–1.544) ***	1.619 (1.563–1.677) ***	
Yes	3711	828	29,196.51	28.36	981	275	7291.67	37.71	1.345 (1.173–1.541) ***	1.265 (1.033–1.549) *	
Co-Morbidity											0.0083
No	47,533	1799	440,522.01	4.08	11,731	601	107,686.96	5.58	1.368 (1.247–1.500) ***	1.668 (1.401–1.985) ***	
Yes	73,206	18,222	492,233.58	37.02	18,453	6483	112,832.69	57.46	1.553 (1.510–1.598) ***	1.606 (1.550–1.664) ***	
Co-Medication											0.3858
No	82,556	8347	713,267.74	11.70	20,510	3009	170,702.02	17.63	1.510 (1.448–1.574) ***	1.641 (1.548–1.739) ***	
Yes	38,183	11,674	219,487.84	53.19	9674	4075	49,817.63	81.80	1.543 (1.489–1.599) ***	1.582 (1.515–1.652) ***	
Urbanization											0.5655
1 (high)	64,110	9427	502,063.88	18.78	15,649	3234	116,125.46	27.85	1.482 (1.424–1.542) ***	1.647 (1.564–1.733) ***	
2	45,504	8227	346,514.94	23.74	11,512	2971	83,443.88	35.60	1.500 (1.438–1.564) ***	1.586 (1.503–1.673) ***	
3	9310	1970	70,591.85	27.91	2569	732	17,973.31	40.73	1.457 (1.338–1.586) ***	1.492 (1.336–1.668) ***	
4 (low)	1815	397	13,584.91	29.22	454	147	2977.00	49.38	1.683 (1.392–2.033) ***	2.033 (1.584–2.610) ***	

*: *p* < 0.05, **: *p* < 0.01, ***: *p* <0.001. ^: IR, Incidence Rate, per 1000 person-years. ^$^: Adjusted for age, gender, low income, OPD utility frequency, hospital days, co-morbidity, co-medication, and urbanization. Abbreviation: (AMI), Acute Myocardial Infarction; (CI), Confidence Interval; (HR), Hazard Ratio; (OPD), Out Patient Department; (PY), Person-Year.

**Table 5:** Sensitivity analysis for the risk of AMI between scabies and non-scabies groups.

Variables	Obs.	AMI (*n* = 3565)			Crude HR (95% CI)	Adjusted ^$^ HR (95% CI)
		Event	PY	IR ^		
Scabies (diagnosed by dermatologists)						
No	127,045	2394	968,708.91	2.47	ref.	ref.
Yes	23,878	465	176,520.49	2.63	1.066 (0.975–1.167)	1.200 (1.063–1.356) **

**: *p* < 0.01. ^: IR, Incidence Rate, per 1000 person-years. ^$^: Adjusted for age, gender, low income, OPD utility frequency, hospital days, co-morbidity, co-medication, and urbanization. Abbreviation: (AMI), Acute Myocardial Infarction; (CI), Confidence Interval; (HR), Hazard Ratio; (PY), Person-Year.

**Table 6:** Sensitivity analysis for the risk of all-cause mortality between scabies and non-scabies groups.

Variables	Obs.	All-Cause Mortality (*n* = 34,352)			Crude HR (95% CI)	Adjusted ^$^ HR (95% CI)
		Event	PY	IR ^		
Scabies (diagnosed by dermatologists)						
No	127,045	22,413	975,562.17	22.97	ref.	ref.
Yes	23,878	4692	177,713.06	26.40	1.148 (1.112–1.185) ***	1.362 (1.320–1.406) ***

***: *p* < 0.001. ^: IR, Incidence Rate, per 1000 person-years. ^$^: Adjusted for age, gender, low income, OPD utility frequency, hospital days, co-morbidity, co- medication, and urbanization. Abbreviation: CI, Confidence Interval; HR, Hazard Ratio; PY, Person-Year.

## Data Availability

Data are available from the National Health Insurance Research Database (NHIRD) published by Taiwan National Health Insurance (NHI) Bureau. Due to legal restrictions imposed by the government of Taiwan in relation to the “Personal Information Protection Act”, data cannot be made publicly available. The Longitudinal Generation Tracking Database 2005 (LGTD2005) was used for this study. There were about 2 million individuals randomly sampled from the Beneficiaries of the National Health Insurance Research Database (NHIRD); the latter comprised approximately 23.75 million individuals in NHIRD. The detail of LGTD2005 please visit the website: https://nhird.nhri.org.tw/en/Data_Subsets.html (accessed on 31 December 2021).
